# 3D Visualization of Body Motion in Speed Climbing

**DOI:** 10.3389/fpsyg.2020.02188

**Published:** 2020-09-28

**Authors:** Lionel Reveret, Sylvain Chapelle, Franck Quaine, Pierre Legreneur

**Affiliations:** ^1^LJK, University Grenoble Alpes, CNRS, INRIA, Montbonnot-Saint-Martin, France; ^2^FFME, Fédération Française d'Escalade et de Montagne, Paris, France; ^3^GIPSA, University Grenoble Alpes, CNRS, Saint Martin d'Hères, France; ^4^LIBM, University Lyon 1, Lyon, France

**Keywords:** speed climbing, video analysis, biomechanics, motion analysis, 3D visualization, center of mass

## Abstract

Speed climbing involves an optimization of the velocity of the ascent and the trajectory path during performance. Consequently, any amount of energy spent in the two other directions than vertical, namely the lateral direction and the direction perpendicular to the wall plane, is a potential loss of performance. To assess this principle, we present a study on 3D motion analysis and its 3D visualization for a subject during a speed climbing performance. The fundamentals of geometrical measurement in 3D require to integrate multiple 2D cues, at least two, in order to extract 3D information. First results with two drones following an athlete's ascent show that a 3D velocity profile can be provided from the tracking of a marker on the harness, pointing critical phases in the ascent where the vertical speed is not dominant any more. We further investigate 3D motion of full body using markerless video-based tracking. Our approach is based on a full body 3D avatar model of the climber, represented as a 3D mesh. This model and its deformation are learned in a laboratory studio. The learning needs to be done only once. Result is a manifold embedding of the 3D mesh and its deformations, which can be used afterwards to perform registration onto video of performance of speed climbing. The results of the tracking is an inference of the 3D mesh aligned onto videos of speed climbing performance. From this 3D mesh, we deduce an estimation of the center of mass (COM). We show that this estimation from 3D mesh differs from the usual approximation of the COM as a marker on the harness. In particular, the 3D mesh COM takes into account the whole body movement such as the influence of the limbs which is not detected by a marker on the harness.

## 1. Introduction

Video analysis is now regularly used by high-level athletes and coaches to address performance optimization. Sequences are usually acquired through portable devices or fixed environment, allowing to quickly visualize a current trial and providing an instant feedback on the performance. The quantification of benefit of such video feedback in self-control condition has been reported in Basketball shot for example (Aiken et al., 2012) and specifically in climbing (White and Olsen, 2010). While some angle of view are naturally preferred, typically dorsal view in our case of interest about speed climbing, it logically provides a 2D view only of the performance. One might consider this as a limitation since the range of motion of the performance is deeply embedded in 3D. One straight-forward solution is to use additional viewpoints such as a side view to better assess the tri-dimensionality of motion. This raises the problem of presenting the user with several “windows” which may lead to overwhelming information spread over all views. We present here an original approach to automatically visualize a speed climbing performance in 3D through: (i) the geometrical reconstruction of the climbing scene and (ii) markerless video tracking of a 3D avatar of the performance of the athlete. As an end-result, an interactive 3D scene can be manipulated by non-technical user to inspect all aspect of motion within a single window interface.

Previous works on climbing motion rely wether on 2D video analysis only or inertial measurement unit (IMU) as depicted in the literature review by Orth et al. (2017). Among first kinematical studies of climbing, Cordier et al. introduced the concept of entropy in climbing, based on the convex hull span by the trajectory of a marker on the harness (Cordier et al., 1993). For climbing motion, Seifert et al. stressed the importance of addressing motor-control issues in 3D (Seifert et al., 2014, 2015). From jerk data, calculated using 3D linear accelerations and body orientation, they defined the concept of fluency which can account for the performance of climbers. For 3D position, it is well-known that it is not reliable to deduce this quantity from 3D acceleration due to the propagation of noise in the double numerical integration of the signal. Our goal is to tackle a biomechanical quantity such as COM (Sibella et al., 2008; Zampagni et al., 2011), motivating our focus on the body as a 3D volume. Assessment of the 3D location of COM in climbing has been reported by Sibella et al. (2008). The data were collected from a markers-based system and limited to a 3 m high wall. Such an experimental approach is difficult to adapt for speed climbing, motivating a markerless video-based technique. With the recent advances of Machine Learning approaches, numerous techniques exist now for video-based 3D motion analysis. All of them target a general purpose application with huge learning sets. We focus on a specific athlete for which we built a dedicated 3D biomechanical twin, or avatar. Using this model, we adapted one of our previous work on manifold learning of 3D body shapes in motion (Duveau et al., 2012) to speed climbing gesture.

We describe here a method to capture 3D information about the motion of a speed climbing athlete from video acquired by several points of view, fixed, or possibly moving such as drones. The key aspect of our method is to be based on a 3D avatar of the athlete. This avatar is first learned in a laboratory studio. Granting the morphology of the athlete is not significantly changing, this learning needs to be done only once. Afterwards, the learned 3D avatar is automatically registered onto any video of the performance of the athlete, without the need for any markers. The end result of the approach is an animated 3D representation of the performance which can be interactively explored by changing viewpoint. In addition, we show that a prediction of the 3D trajectory of the COM can be derived from this 3D representation, providing an estimation more reliable than a marker on the harness.

## 2. Methods

### 2.1. Calibration of Viewpoints

In video recording of climbing performance, viewpoints from the ground classically introduce artifacts such as bottom view distortion which impedes the efficiency of visual inspection and automatic processing. For this experiment, we have thus used two drones as they provide high-quality video capabilities and can be easily monitored to follow the ascent of the athlete. As each drone is moving with respect to the environment, motion recorded in the video mixes both the motion of the athlete and the motion of the drone. This is resolved by performing an auto-calibration of the drones 3D position and orientation at each frame with respect to reference frame linked to the wall. Drones usually embed inertial and GPS sensor to monitor their position. Such sensors turned out to be not precise enough to compute an accurate estimation of their relative position and orientation—accuracy goal is to be <1 cm and 1 degree per frame). We used instead a geometrical approach, based on a prior 3D scanning of the wall. At each frame, a prior 3D model of the wall is registered onto the video view by aligning salient features of the holds. Registration is performed by optimizing the 3D location and orientation of the drone with respect to a metric on wall features. Like for traditional markers-based system, the quality of such a calibration can be assessed through back projection. Results showed that the required accuracy can be achieved.

### 2.2. Extraction of the 3D Trajectory of a Marker

As a first result following calibration and as a matter of validation, we implemented a 3D reconstruction of the trajectory of a marker on the harness. While not exactly the COM (as detailed later), such a location is close enough to COM to be worth noticing and to be considered as a good representative of the overall body 3D location. The 2D location of the marker is tracked on each view using normalized cross correlation. As the 3D location and position of each drone is known at each frame, the 3D location of the marker can be derived using Direct Linear Transform approach (DLT). Drones are calibrated with respect to a fixed reference frame related to the wall, hence the extracted 3D location of the marker. By derivating this 3D location using finite difference and knowing the video frame rate, the athlete's speed can be estimated in metric units. This constitutes a first visualization of a motion quantity in 3D, with a possibility to identify key moment where the vertical speed is decreasing or when the climber is getting too much away from the wall, inducing a loss of performance for the goal of reaching the top in minimal time.

### 2.3. Construction of the 3D Avatar

To go beyond the 3D trajectory of an isolated marker, we focus now on 3D visualization of the body as a whole based on a 3D mesh representation. This 3D representation will be used to implement a markerless video tracking approach (next section). To build this 3D model of the athlete, we used a laboratory facility consisting in a studio equipped with 68 video cameras. This studio allows to compute, for each video frame, a full 3D reconstruction of the body surface in motion using a convex hull approach (Laurentini, 1994). The process consists in, first, calibrating the video camera so that their location and orientation in 3D space are known. During the live performance, the silhouette of the subject is segmented using background substraction. From this silhouette, a 3D generalized cone is computing for each camera view, made of the camera location at the apex and the silhouette at the base. Finally, the geometrical 3D intersection of all the cones provides the resulting 3D surface of the subject. Unlike a range scanner, such an approach does not deliver the exact 3D surface but an approximating tangent hull. However, with as many as 68 cameras, it can be considered that the convex hull is closely approaching the true 3D surface within a sub-millimeter accuracy. Having a 3D surface allows to easily derive an estimation of the COM using geometrical computation, under the assumption of a constant density of 1 *kg*/*dm*^3^. This experimental data provides the required information to learn the manifold of all the deformations of the athlete's 3D body surface. This learned model can subsequently be used for tracking live motion from video during a performance on the wall.

### 2.4. Automatic Tracking of Whole Body 3D Motion From Video

It cannot be envisioned to deploy a set-up with 68 fixed cameras or 68 drones at the speed climbing wall for a day-to-day practice. Instead, we use the set of 3D meshes in motion to learn a manifold of 3D shapes of the athlete. The tracking procedure consists in registering the 3D mesh model of the athlete onto the two drones video view by optimizing the manifold parameters with respect to salient body features (Duveau et al., 2012). The manifold allows for a reduction in dimensions which guarantees the convergence of the registration. At the end of this stage, an instance of the 3D surface of the athlete is inferred onto video, providing a 3D encoding in the frame reference of the climbing wall. Using a registration on two views prevents from ambiguities and occlusions and guarantees a better fit between the 3D model and the real pose of the athlete during ascent.

One female climber performed a set of maximal speed ascents on the official route. We selected the best trial (ascent time = 7.9 s) for this analysis. The performance has been filmed with two drones (DJI Mavic pro) with resolution 3,840 *x* 2,160 pixels at 30 fps. Drones are following a purely vertical ascent, at a distance of about 8 m from the athlete (one pure dorsal view, one apart from 45 degrees angle). The two drones have been temporally synchronized at the frame level with a common light signal triggered at the beginning of the performance. The procedures (data collection and analysis) were approved by the French Federation of Mountaineering and Climbing (FFME), and conformed to the declaration of Helsinki. It has been approved by the University of Lyon ethic committee as not invasive because it is limited to the video recording of a regular practice without any contact with the subject's body. Piloting of the drones was performed under the supervision of a certified pilot (drone certificate MAVIC-53) in a closed non-public environment. Flying was limited to a vertical ascent of <20 m.

### 2.5. Assembling the 3D Scene

We assemble all the results into a final 3D scene. First, the 3D scan of the wall used for calibration can be directly imported. Dedicated texturing can be added to augment the quality of the rendering. The 3D mesh representation of the athlete in motion is integrated, with also a possible texturing for aesthetics consideration. It is worth noticing that such texturing needs to be done only once off-line as the topology of the mesh remains constant. Only the 3D location of the vertices is updated by the automatic tracking phase. For the sake of validation of the process, the original video of the drones can be first augmented with the projection of the 3D animated scene (**Figure 2**). The 3D animated scene can also be explored from 3D viewpoint, different from the original drone video viewpoint, following a subjective camera principle (**Figure 3**). Lastly, the 3D view can be augmented with information such as the 3D trajectory of the COM or velocity cues. Hints on time of grasping of the holds can also be visualized by computing 3D velocity of mesh vertices at limbs extremities.

## 3. Results

### 3.1. Subject Trial

**Figure 1:**
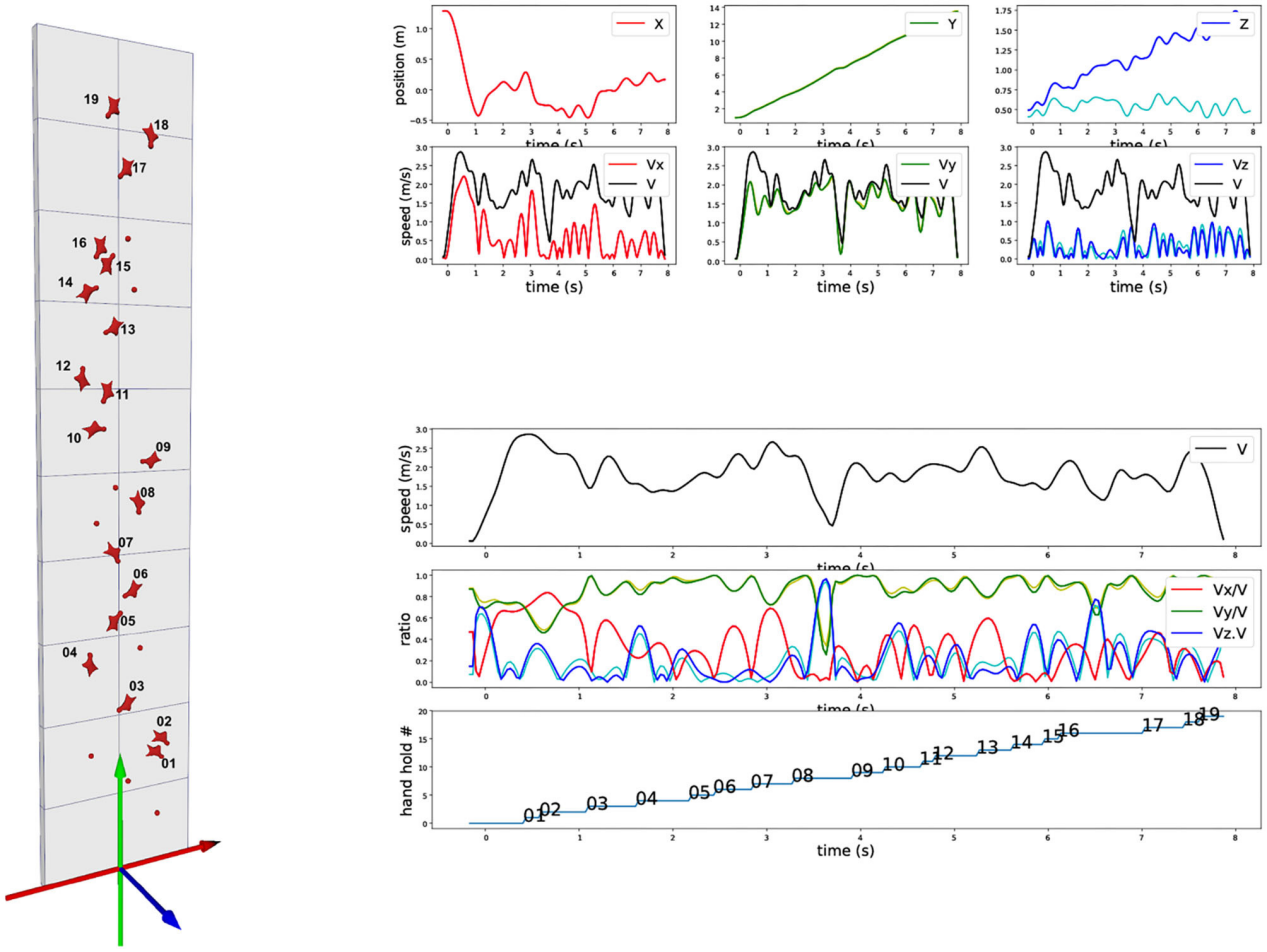
Trajectory and velocity of a marker on the harness. (Left) Standard speed climbing wall with lateral axis (X-red), vertical axis (Y-green), and axis perpendicular to XY plan (Z-blue). (Top right): (upper) 3D trajectory of XYZ components over time of a marker on the harness (dark blue is the world-space Z-trajectory, light blue is the Z-trajectory with the cancelation of the 5 degrees inward inclination of the wall, corresponding to the exact perpendicular distance to the wall); (lower) corresponding XYZ velocity components. (Bottom right): (upper) Velocity euclidian norm across time; (middle) XYZ velocity components ratios with respect to the velocity euclidian norm; (lower) Vertical progression of the marker with respect to the hold numbers as represented on the left.

Center of mass of the subject has been first approximated by a marker attached to her harness, close to the middle of the pelvic ilium bones. The 2D trajectory of this marker has been digitized on each video using image normalized correlation. The 3D reference frame is made of the horizontal ground (plane XZ) and the gravity vertical axis (axe Y), with the origin at the bottom of the wall (Figure 1). Triangulation provides the 3D trajectory of this marker into this world reference frame. This paper presents the methodology of the 3D visualization and focuses thus on a single trial.

### 3.2. 3D Trajectory of the Harness Marker

We report on Figure 1 the three components of the 3D position of the marker (first row). Data have been processed with a low-pass Butterworth filter (order 5, cut-off frequency 5 Hz). X direction is lateral, Y direction is vertical, and Z direction is perpendicular to the wall minus 5 degrees because of the wall inclination. We also report the corresponding velocity component for this marker in m/s (second row) and the overall 3D norm of the velocity (black curve, identical on each plot). As the trajectory is measured in 3D in world reference frame (gravity exactly vertical), the 5 degrees inclination of the wall appears but can be easily canceled out. This is of particular interest for the Z direction, as such a cancelation allows to clearly visualize the distance of the climber with respect to the wall. Figure 1 shows both the results in the world reference frame (red, green, and blue) and in the wall reference frame after wall inclination correction (magenta, yellow, and cyan). Biggest difference is on the Z-component, with of course no impact on the overall speed norm. Red and magenta curves are exactly overlapping as the two reference frames differs only in X-axis rotation.

On bottom of Figure 1, we explore the ratio of the velocity components with respect to the total velocity. First row is a recall of the total velocity, second row the three velocity component ratios together, and the last row indicates the evolution of the ascent with respect to “hand” holds number (we omit “feet” holds for clarity). The curve corresponds to the moments when the hips marker vertical position goes above the holds. The different flat areas thus provide an estimate of the time spent between two holds during vertical ascent.

The velocity ratio clearly outline moments when the vertical ascent is less dominant. It typically corresponds to “dyno” transition from hold 8 to 9 and hold 16 to 17. During these periods, the wall-orthogonal Z-axis component becomes dominant, corresponding to a posture which is getting farther from the wall. On hold 7 to 8 and hold 13 to 14, the velocity components ratios profiles show that the lateral X-axis becomes important with respect to the vertical Y-axis. They correspond to a required change of route but also show a drop in the vertical component. The visualization of these 3D cues provide first insights on speed climbing performance.

### 3.3. 3D Full Body Tracking

**Figure 2:**
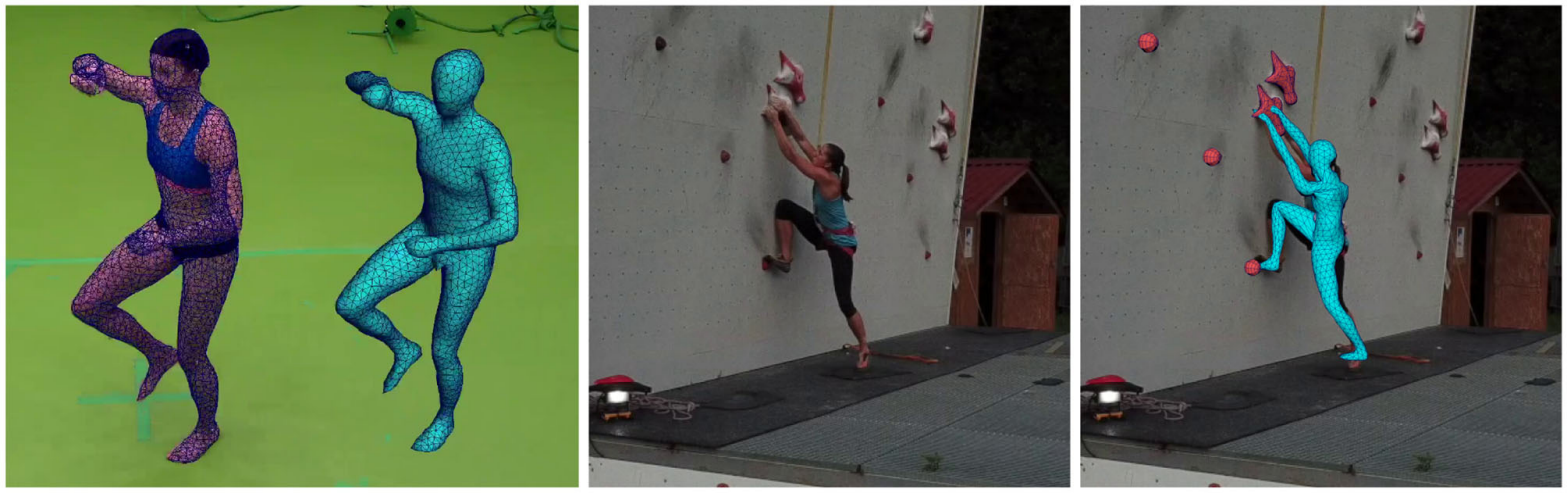
3D avatar of the athlete and its overlay onto video : during training at the lab (left), original test input condition (middle), tracking result overlay (right).

The previous steps validated the experimental infrastructure to extract 3D information from video in terms of the trajectory of a single marker. We report here the extension to full body analysis through its 3D visualization. The official 15 m high speed climbing wall can obviously not be replicated into the laboratory as the volume which can be captured at the laboratory facility is limited to 5 × 5 × 3 m. Consequently, the athlete has been asked to perform a mimicry of the speed climbing ascent as the route is standardized and completely memorized. Protocol for the simulation of movement has been left to the expertise of the climber who is a word-level athlete. We used this sequence to learn the manifold of 3D shape variation of the athlete's 3D surface appearance. We used a Gaussian Process Latent Variables Model as described in Duveau et al. (2012). Simulation of motion in laboratory conditions has been used to bootstrap the prediction algorithm through the machine learning phase. It does not impose the real wall condition to exactly replicate the motion in laboratory as some generalization is allowed. Joint angles start from the configuration collected in laboratory but are modified in value and timing to fit the real world case using model alignment. Figure 2 on the left shows the model learned in the laboratory and the result of the automatic registration onto a video from a drone. It is noticeable that the fit between the learned 3D model and the real silhouette of the athlete on the ascent does not match exactly. For the sake of robustness and prevent from drifting in tracking, we currently constrain the model to stay very closed to the learned manifold in the laboratory. The difference between 3D motion during learning and 3D motion during real ascent at the wall explains the local mismatch. Future works include allowing more degrees of freedom in the model so that local features such as feet and hands orientations can be better recovered. As for now, we focus only on the overall 3D motion of the body. In particular, we explore here the prediction of the COM from the 3D mesh on which local adjustments of feet and hands will not have a significant impact.

### 3.4. COM Estimation : Marker on the Harness vs. Prediction From 3D Mesh

**Figure 3:**
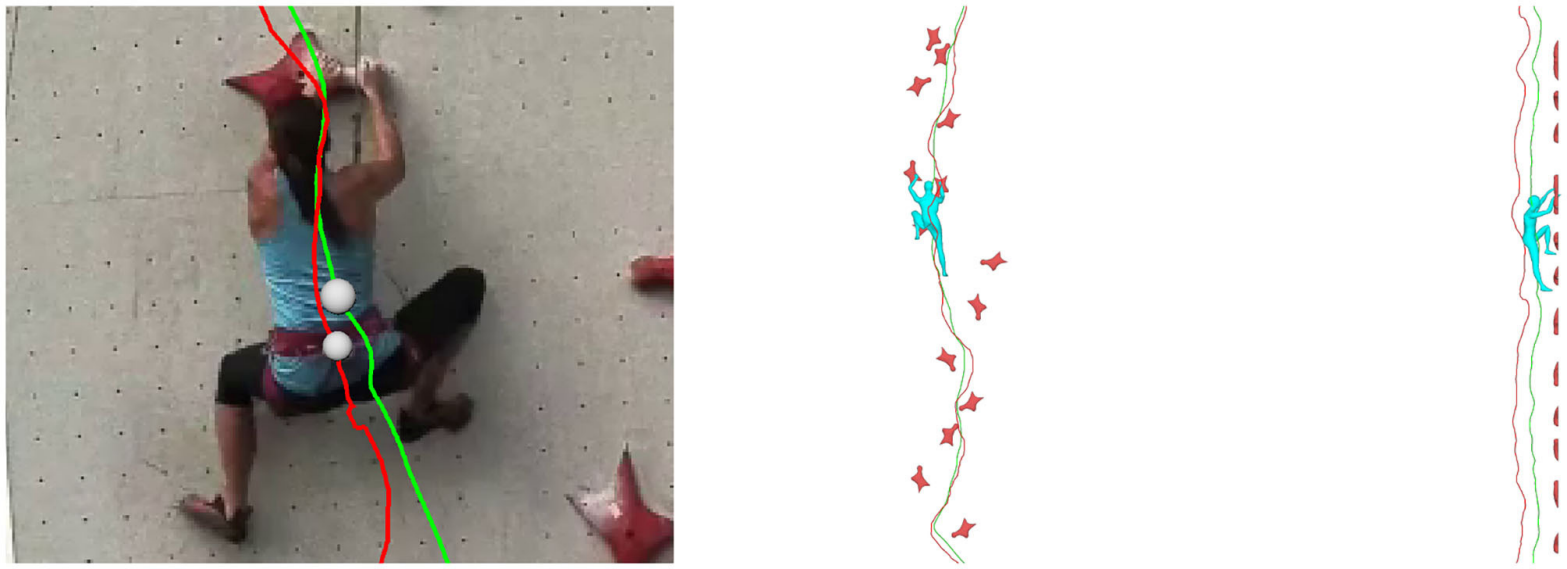
Comparison between trajectories from marker on the harness (red curve) and COM computed from 3D mesh (green curve).

We examined here the approximation of considering the COM as a fixed marker on the harness near the hips vs. a prediction as the COM of 3D mesh. The prediction from 3D mesh was automatically computed from the 3D mesh as the barycenter of the enclosed 3D volume with the hypothesis of uniform density. We compared this trajectory to the trajectory of the marker on the harness with the result of this prediction. Results show that the mean distance between the marker on the harness and the COM from 3D mesh are, respectively in the three directions: 7.3 ± 5.9 cm for the X direction (lateral), 8.7 ± 4.6 cm for the Y direction (vertical), and 24.1 ± 4.3 cm for the Z direction (perpendicular to wall). The biggest difference is on the Z direction as the marker is attached on the back while the 3D mesh COM is more likely also influenced by limbs projection toward the wall. Figure 3 illustrates on overall comparison between the trajectory of the marker and the trajectory of the 3D mesh COM. It shows that the actual trajectory of the 3D mesh COM appears smoother than the trajectory of the marker. We also report a situation where the difference in vertical direction has been reported maximal (3D mesh COM is 16.1 cm above the marker). It corresponds to a case where the legs are flexed into an upward position. This explains well why the 3D mesh COM is actually moving upper than the marker on the harness which thus proves not to be always a good approximation of the true COM.

### 3.5. Visualization of Extra Cues in 3D

**Figure 4:**
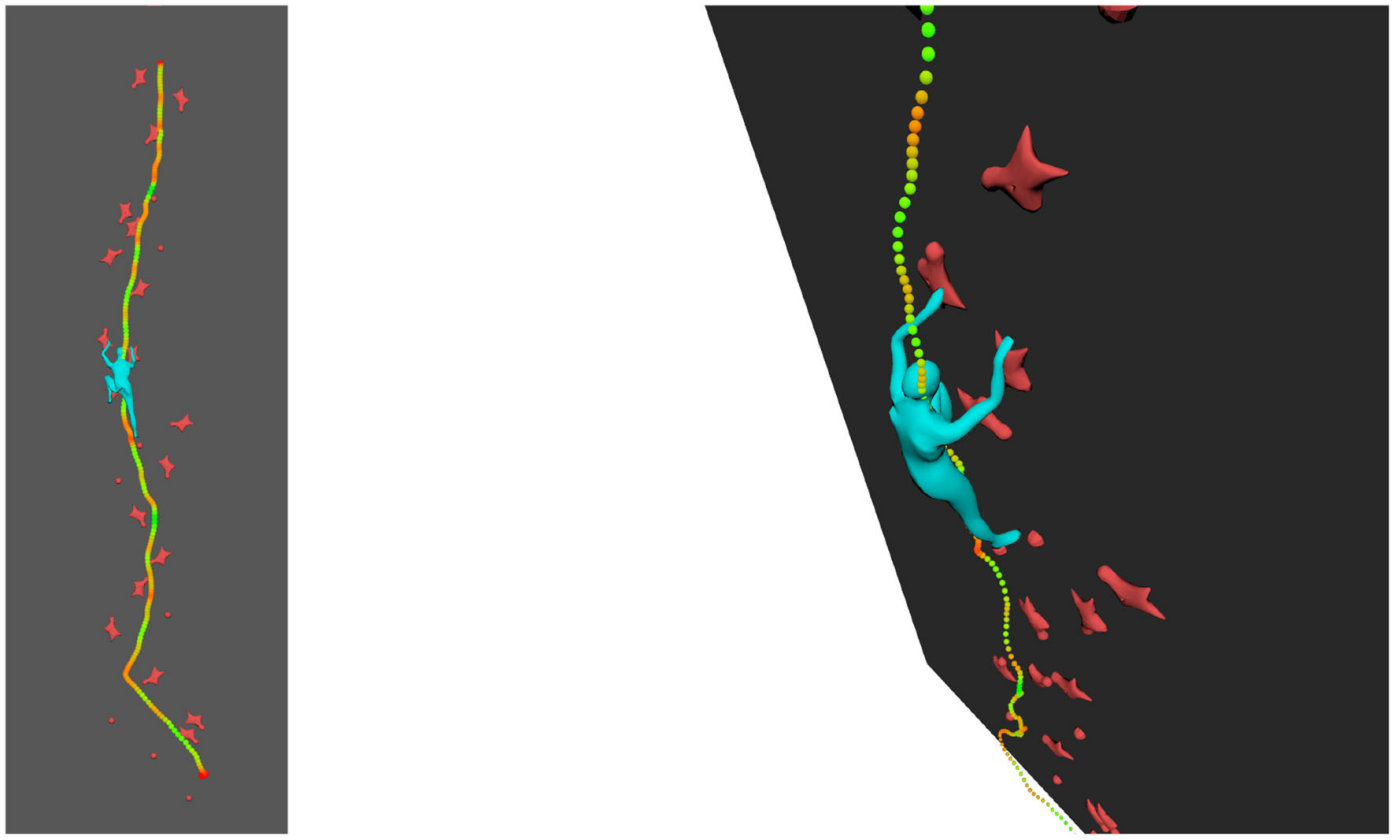
Velocities with color ramp related to velocity magnitude (minimal-red to maximal-green).

The 3D scene can be visualized from different angles and as such represent a valuable enhancement of standard video. In addition, visual cues can be added onto the 3D scene such as velocity. In Figure 4, all the position of the COM have been reported during ascent, with a color ramp associated with magnitude of velocity ranging from minimal velocity in red to maximal velocity in green. Other visual cues can be integrated into the 3D view.

## 4. Discussion

**Figure 5:**
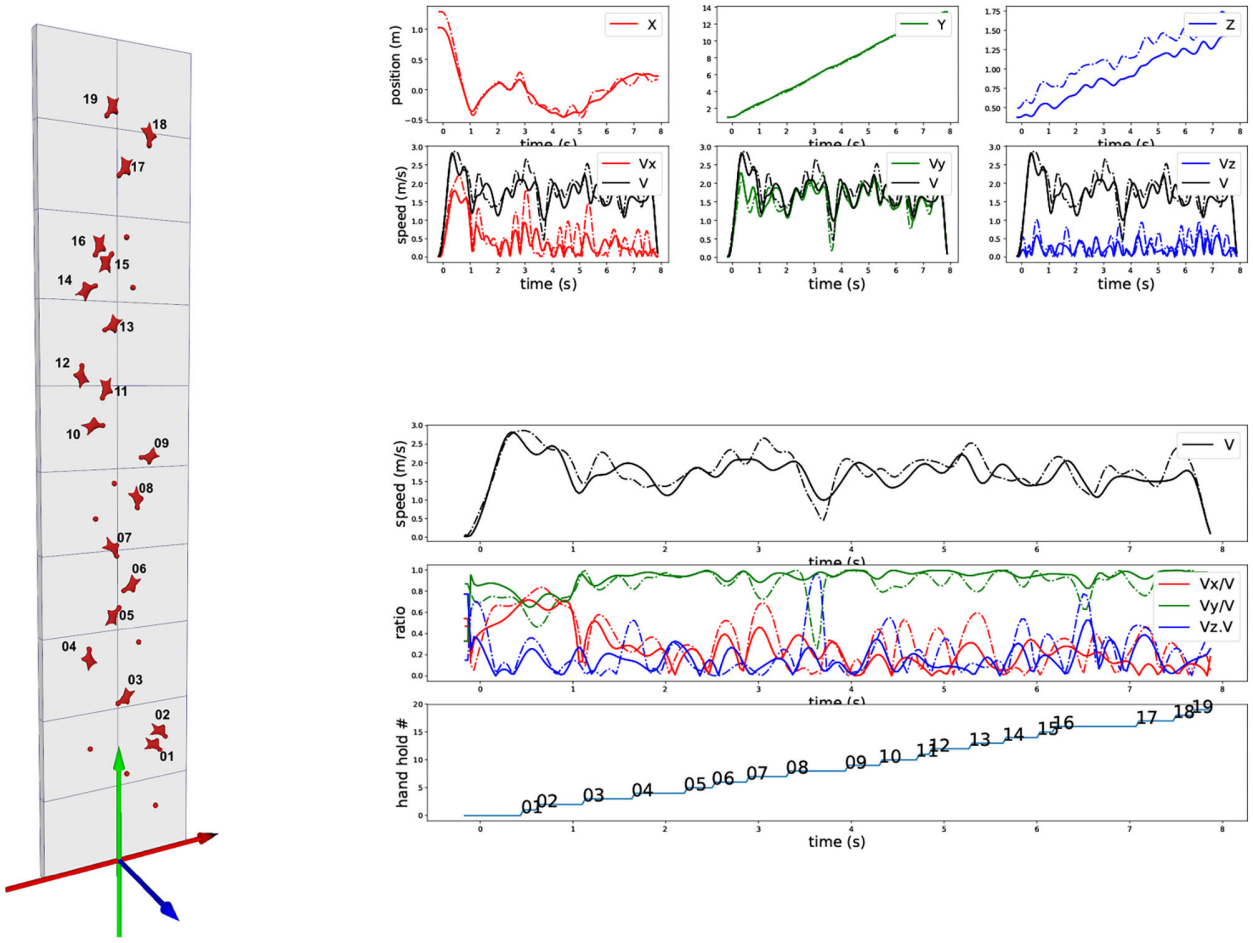
Comparison of trajectories and velocities between marker on the harness (dashed line) and COM computed from 3D mesh (plain line). Curves correspond exactly to the same layout as in Figure 1: Top-right are XYZ trajectories and velocities, Bottom-right are velocities and vertical progression (see caption of Figure 1 for details).

Results show that the trajectories of a marker on the harness and the 3D mesh COM differ. Although no exact measurement of the true COM exists for validation, its estimation from the 3D mesh follows some rational insight and tends to prove it is more reliable. Typically, unlike the marker, the 3D mesh method accounts for the projection of the limbs toward the wall or the flexion of the legs. Therefore, we compared the velocity computed from the marker with the one computed from the COM, following the same scheme as Figure 1. Results in Figure 5 show significant differences, especially around the “dyno” section when body crosses hold 08. Trajectory of the marker is displayed in dashed line and trajectory of 3D mesh COM is in plain line. After a close look at this section, it confirms that the COM from 3D mesh provides a more meaningful interpretation with respect to the true COM, compared to the marker. Indeed, at this section, even if the pelvis is actually stopping, inducing a loss of vertical speed of the marker, the legs are still moving upward. Similarly to the case reported on Figure 3, it suggests why the true COM maintains an upward velocity and why the 3D mesh COM is a better estimate.

**Figure 6:**
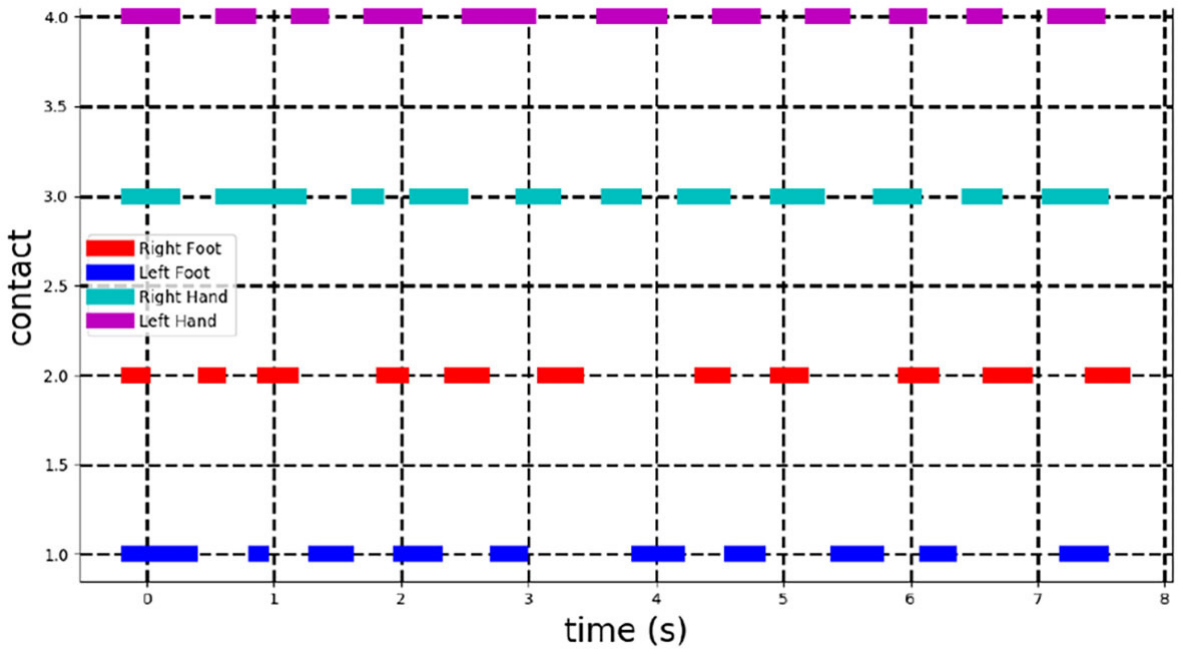
Detection of contacts on the holds and the wall. Each line corresponds to one of the body limb extremity (right foot, left foot, right hand, left hand). It is plain when a contact is detected. A contact is assumed to be hold when velocity of its corresponding vertex on the 3D mesh is below a threshold of 1*cm*/*s*.

Video-based technology is a promising alternative to makers-based system because of its practical use. It is however difficult to exactly evaluate its accuracy against the later approach unless both set-up, with and without markers, are installed at the same location. However, the experiment presented here shows the potential of the approach, first for a qualitative feedback through 3D visualization and also, for quantification of high-level features such as an estimation of the 3D trajectory of the COM. As an example of complementary features extraction and visualization, the 3D tracking allows to measure valuable cues on the timing of holds grasping. Indeed, by inspecting the velocity of the mesh vertices at hands and feet, a duration of the grasping can be deduced. Figure 6 shows the result for this ascent with a threshold of 1 *cm*/*s* to identify grasping from velocity magnitude of limbs extremity vertices (Figure 6). In this case, a user evaluation can be performed as the extracted information is binary, on or off, and is visually identifiable. Results revealed that the identification of grasping duration is perfectly accurate and even include cases when the athlete is using contacts with parts of the wall outside hands and feet holds.

## 5. Conclusion

In the absence of side-by-side experiments with both equipments, it cannot be claimed that a markerless video-based method reaches yet the accuracy of a standard markers-based systems. However, we show that our technique provides reliable cues such as a usable 3D visualization of the whole body in motion and estimation of the 3D trajectory of the center of mass (COM). In particular, our finding is that they are some noticeable discrepancies between the 3D trajectory of a marker on the harness, approximating the COM, and an estimation of the COM of the 3D model of the athlete registered on videos. The estimation of the 3D trajectory of the COM from our video-based 3D mesh tracking tends to follow rational insights that a marker-based approach does not allow.

Future works will explore more precisely dynamics in speed climbing. The goal will be to extend our previous experience in this domain, obtained in a laboratory set-up, to similarly address the context of athletic speed climbing (Quaine and Vigouroux, 2004). In particular, following the markerless objective, we will continue to adapt our previous approaches for prediction of contact forces from kinematical data only (Quaine et al., 2017) through numerical optimization.

## Data Availability Statement

The datasets presented in this article are not readily available due to ethical concerns regarding confidentiality. Requests to access the datasets should be directed to Lionel Reveret and Sylvain Chapelle.

## Ethics Statement

The studies involving human participants were reviewed and approved by University Lyon 1—Ethics committee. The patients/participants provided their written informed consent to participate in this study. Written informed consent was obtained from the individual(s) for the publication of any potentially identifiable images or data included in this article.

## Author Contributions

LR developed the techniques, performed the experiment to collect the data to build the athlete's 3D model, performed the 3D video analysis, computed the 3D model kinematics, and wrote the paper. SC provided the sport supervision. PL performed the experiment during the speed climbing tests. FQ and PL participated in the writing of the paper. All authors contributed to the article and approved the submitted version.

## Conflict of Interest

The authors declare that the research was conducted in the absence of any commercial or financial relationships that could be construed as a potential conflict of interest.
